# Comparative Diagnostic of Cervical Tuberculous Lymphadenitis: PCR is a Fast, Efficient, and Improved Diagnostic Approach

**DOI:** 10.1155/2023/3312250

**Published:** 2023-06-13

**Authors:** Himanshu Jha, Chandra Prakash Baveja, Vinay Kamal, Prem Narayan Agarwal, Sonal Saxena, Megh Singh Dhakad, Divakar Sharma

**Affiliations:** ^1^Department of Microbiology, Maulana Azad Medical College, New Delhi 110002, India; ^2^Lab Strengthening & Diagnostics, Jhpiego, India; ^3^Department of Pathology, Maulana Azad Medical College, New Delhi 110002, India; ^4^Department of Surgery, Maulana Azad Medical College, New Delhi 110002, India; ^5^Department of Microbiology, Lady Hardinge Medical College, New Delhi 110001, India

## Abstract

**Methods:**

The study included 100 clinically suspected cases of TBLN. Fine needle aspirate (FNA) samples were processed for cytology staining and cultured on LJ & BACTEC 12B media. The biochemical tests were performed to identify the isolates at the species level. Additionally, for PCR, DNA was extracted and used for the diagnosis and identification of mycobacterial species.

**Results:**

Patients ranged from 2 to 45 years with a mean age of 24.96 ± 9.10 years. Out of 100 patients, 73% had clinical symptoms of weight loss, followed by fever (72%), anorexia (66%), and night sweats (58%). 24% of patients were found to be smear-positive after Ziehl–Neelsen (ZN) staining and statistically highly significant with PCR. On LJ medium 34% and on BACTEC radiometric 45% of samples were smearing positive. Overall, 48% of cases were PCR-positive for TBLN. When compared with culture, the sensitivity and specificity of PCR were 93.75% and 100%, respectively, which are higher than cytology. The true positive predictive value (PPV) and negative predictive value (NPV) were 83.3% and 61.5%, respectively.

**Conclusion:**

This study suggests that PCR is a rapid, sensitive, and specific tool for correct diagnosis of TBLN cases as compared to staining and culture which lead to the early and proper management of mycobacterial diseases.

## 1. Introduction

Mycobacterial infections have mediated diseases reported globally, but are particularly more prevalent in developing countries such as South-East Asia (45%), Africa (23%), and the Western Pacific (18%). According to the World Health Organization (WHO) report (2022), 10.6 million people were ill and 1.6 million people lost their lives due to TB all over the world [1]. According to a WHO report, eight countries accounted for more than two thirds of global TB cases: India (28%), Indonesia (9.2%), China (7.4%), the Philippines (7.0%), Pakistan (5.8%), Nigeria (4.4%), Bangladesh (3.6%), and the Democratic Republic of the Congo (2.9%). India has the highest TB burden, accounting for 28% of the global incidence [1, 2]. Still, TB remains a major cause of death among infectious diseases.

Although the predominant form of tuberculosis is pulmonary TB disease; however, infection with *M. tuberculosis complex* may be seen in any organ of the body. Extrapulmonary tuberculosis (EPTB) is defined as the isolated occurrence of TB in any part of the body other than the lungs [3, 4]. Tuberculous lymphadenitis (TBLN) is the most common clinical presentation of extra-pulmonary tuberculosis [5]. Cervical lymph nodes are the most common sites and are reported in 60% to 90% patients with or without involvement of other lymphoid tissue [6].

Early confirmatory diagnosis of tuberculosis is a challenging especially in case of paucibacillary and extra-pulmonary forms. Conventional methods available for the diagnosis have limitations. Histopathology is characteristic but there could be problems to get representative specimen, and nonspecific features. In tubercular lymphadenitis cases, a rapid, sensitive, and specific diagnosis is needed owing to the limitations of the traditional microbiological methods, the paucibacillary nature of the specimen, and the extensive differential diagnosis [7].

Laboratory diagnosis of tuberculosis is based on the traditional methods of Ziehl-Neelsen acid-fast stain and laboratory culture of the causative organism. Despite its usefulness in the diagnosis of TBLN, fine needle aspiration cytology (FNAC) faces several limitations, and its sensitivity and specificity are not well established [8].

Culture on LJ medium is the gold standard, but it takes 8–12 weeks, requires approximately 10–100 bacilli in the sample [6, 9]. The radiometric method (BACTEC) is being used for early diagnosis of tuberculosis which takes lesser time approximately 11-12 days. Data from various studies indicates that BACTEC is considered as a promising method for the rapid detection of mycobacterial growth; however, this system should be used concurrently with conventional culturing methods [10].

The QuantiFERON-TB Gold test (QFT-G) is a whole-blood test for use as an aid in diagnosing *Mycobacterium tuberculosis* infection, including latent tuberculosis infection (LTBI) and tuberculosis (TB) disease [11]. The results of this assay are based on the amount of IFN-gamma released in response to the antigens (ESAT-6 and CFP-10). This assay neither boosts responses like tuberculin skin tests nor is affected by BCG (bacille Calmette-Guérin) vaccination.

Polymerase chain reaction (PCR) has enhanced the diagnostic predictability of the disease, especially in extrapulmonary and paucibacillary samples. The PCR is a rapid diagnostic and data is available in the same day as DNA extraction from the sample [12]. Developments in this area have been very rapid, and a large number of PCR assays targeting different gene stretches of *M. tuberculosis* have been described [13]. The system offers the ability for mycobacteriology laboratories to identify many of the recently described mycobacteria. Furthermore, PCR technology is simple and easy to implement in most of the laboratories. In the present study, we have used various diagnostic techniques, i.e., cytology, hematology, culture, and polymerase chain reaction (PCR) to diagnose clinically suspected cases of TBLN.

## 2. Materials and Methods

This comparative study included one hundred (*n* = 100) clinically suspected cases which were suggestive of TBLN by cytopathology. Fine needle aspirates (FNA)/pus samples from the lymph nodes of these patients were collected at a 1500 bedded tertiary care hospital and processed in the Department of Microbiology, Maulana Azad Medical College, Delhi.

### 2.1. Ethics Approval

Ethics committee approval was granted by the Institutional Ethics Committee of the College and Associated Hospitals, India, and the Ethical approval number is F.501(134)/EC/06/MC(Aca.)197. Written consent was taken from the patients, and they were informed that their participation was voluntary and that they could withdraw from the study at any stage without incurring any penalty.

### 2.2. Collection and Processing of Samples

Fine needle aspirates (FNA)/pus from the lymph nodes collected aseptically from all the adult cases in sterile containers and transported quickly to the mycobacteriology laboratory. The specimens were decontaminated by N-acetyl L-cysteine sodium hydroxide (NALC-NaOH) method [14]. Decontaminated samples were used for diagnostics by conventional methods.

### 2.3. Conventional Methods

#### 2.3.1. Ziehl-Neelsen Staining for Acid-Fast Bacilli

After the decontamination (NALC-NaOH) process, smears were made from all specimens and stained by Ziehl-Neelsen (ZN) staining for acid fast bacilli (AFB) observation. AFBs were observed as red, beaded, and slightly curved rods against a bluish background [14].

### 2.4. Cytology

Giemsa staining was also done for the presence of granulomas, langhan's giant cells, epithelioid cells, and caseous necrosis, which suggest the tuberculosis [15].

### 2.5. Lowenstein-Jensen Medium

The processed fine needle aspirate samples were inoculated on two slants of LJ media and incubated at 37°C. These were examined within 5–7 days for the detection of growth of mycobacteria, any fungal growth, for any contamination and decay of the media. All cultures were examined weekly for 8 weeks, after which they were discarded. As soon as any growth was evident on the culture medium, a smear was made and stained by the ZN stain for confirmation of the acid fast bacilli (AFB) growth. All the growth was then examined for the rate of growth, pigmentation, colony morphology, and other properties [14].

### 2.6. BACTEC 12B Medium (Radiometric Culture)

The sediment of the sample was inoculated into the BACTEC 12B vial medium, along with 0.1 ml of PANTA (mixture of different antibiotics-polymyxin B, amphotericin B, nalidixic acid, trimethoprim, and azlocillin) and incubated at 37°C. Inoculated media were examined in the BACTEC 460 TB system and growth indices (GI) was taken three times a week (after every 2-3 days) for the first three weeks and weekly thereafter for another three weeks (total six weeks). If the GI was greater than 30, then it was considered as positive. As soon as the culture medium showed positive GI, a smear was made and stained with ZN stain for confirmation of AFB [16].

### 2.7. Biochemical Characterization

The colonies were further subcultured on three more slopes of LJ media to identify and differentially confirm *Mycobacterium tuberculosis complex* and nontuberculous mycobacteria (NTM). The different Biochemical tests (niacin production, nitrate reduction, catalase test, urease test, Tween 80 hydrolysis test, aryl sulfatase test, and pyrazinamidase test) were performed to identify the isolates to species level [14]. *Mycobacterium tuberculosis* was taken as a positive control and *Mycobacterium intracellulare complex* as a negative control. The colonies positive for niacin production and nitrate reduction tests and weak positive for catalase tests were labelled as *Mycobacterium tuberculosis* [14].

### 2.8. Molecular Characterization

DNA extraction from FNA/pus samples and from positive *Mycobacterium tuberculosis* cultures was done using the lysozyme and Proteinase K method [17, 18]. Confirmations of isolated DNA were analyzed on a 2% agarose gel, and purity was checked by spectrometer [19]. The DNA was amplified by using Hsp65 primer TB11 (5′-ACCAACGATGGTGTGTCCAT-3′) and TB12 (5′-CTTGTCGAACCGCATACCCT-3′) for the diagnosis and identification of mycobacterial species. With this set of primers, amplification of a 439 base pair region between positions 398 and 836 from the gene (encoding the 65 KDa heat shock protein/hsp 65) was performed in a 25-*μ*l reaction mixture. The reaction mixture for PCR was prepared by adding the reagents as follows: 10 X buffer 2.5 *μ*l; 50 mM MgCl2 2 *μ*l; 10 mM dNTP'S 1.5 *μ*l; primer forward (25 pmol) 1 *μ*l; primer reverse (25 pmol) 1 *μ*l; 5 U of *Taq* DNA polymerase 0.3 *μ*l; DNA template 5 *μ*l; and nuclease free water 11.7 *μ*l.

The amplification cycle was performed by using a thermocycler (BIO-RAD, USA) with the following program: initial denaturation for 5 minutes at 95°C and 35 cycles with the following steps: 1 minute of denaturation at 94°C, 1 minute of annealing at 60°C, and 1 minute of extension at 72°C. The final extension was for 7 minutes at 72°C. The amplicons were analyzed on a 2% agarose gel, visualized under UV, and photographed using the gel documentation system (Alpha Innotech, USA) [18].

### 2.9. Statistical Analysis

The findings of the PCR were correlated with the findings of other diagnostic techniques. Culture was considered as a gold standard for the statistical analysis, i.e., sensitivity and specificity of different assay methods were calculated. The results were analyzed with SPSS version 16. The *p* values <0.05 were considered significant.

## 3. Results

**Figure 1:**
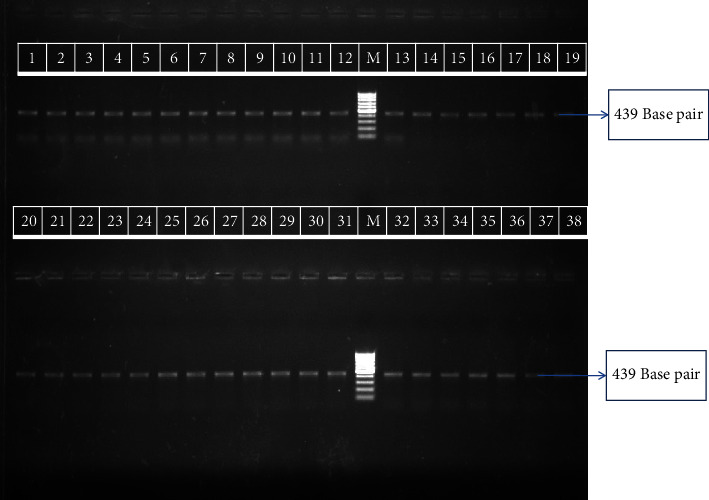
Amplified product of HSP 65 gene at 439 base pair. Lane (M) molecular weight marker100 base pair. Lane 1: standard *M. tuberculosis H*_*37*_*Rv*, lane 2 to 38; all clinical isolates.

All the patients in the study group ranged from 2 to 45 years with a mean age of 24.96 ± 9.10 years. Out of 100 patients, 73% patients had clinical symptom of weight loss, 72% of them had a fever, 66% patients had anorexia, and 58% patients had a complaint of night sweats. In these patients, the most common region of lymph node swelling was the cervical region. Overall 48% cases were PCR-positive by the Hsp 65 gene for TBLN at the 439 base pair region (Figure 1).

**Figure 2:**
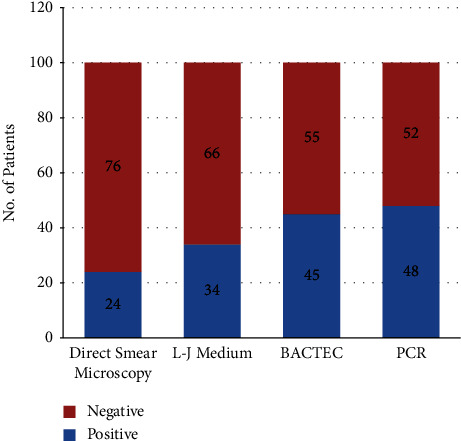
Acid fast bacilli positive by different methods in fine needle aspirates samples (*n* = 100).

24 (24%) samples, which were positive by direct smear microscopy, were also positive by PCR. Among the 76 (76%) specimen samples, which were negative by direct smear microscopy, 24 (31.6%) were positive by PCR (Table 1 and Figure 2). When PCR was compared to direct smear microscopy, the *p* value was observed to be highly significant (*p* value = 0.0001). Thirty-four (34%) of the samples showed growth on LJ medium. 45 (45%) of the samples showed growth in BACTEC culture, and 48 (48%) of the samples were positive by PCR (Figure 2). The 34 (34%) samples which were culture-positive on LJ medium were also positive by BACTEC culture.

There were additional 11 (11%) samples which did not show growth on LJ medium but were culture positive by the BACTEC method (Table 1). There were additional 14 (14%) samples which did not show growth on LJ medium but were positive by PCR. All the positive samples by conventional procedures like direct microscopy and culture {LJ culture (34) and BACTEC culture (45)} were also positive by PCR (Table 1). All the samples which were positive by direct microscopy, LJ medium, and BACTEC culture were also positive by PCR; however, 3 additional samples were also detected positive by PCR.

The sensitivity and specificity of PCR when compared with LJ medium was found to be 100% and 78.8%, respectively, in these cases. The PPV and NPV were found to be 70.83% and 100%. When PCR was compared to LJ culture results the *p* value was observed to be highly significant (*p* value = 0.0001). The sensitivity and specificity of PCR when compared with BACTEC culture was found to be 100% and 94.54%, respectively, in these cases. The PPV and NPV were found to be 93.75% and 100%. When PCR was compared to BACTEC culture results the *p* value was observed to be highly significant (*p* value = 0.0001).

## 4. Discussion

In developing countries like India where the incidence of tuberculosis is highest, TBLN is a cause for 30%–64% of lymphadenopathy, responsible for about 80% of all exclusively extra-pulmonary tuberculosis cases and more than 6% of all tuberculosis cases [20]. The cervical lymph nodes are the most common site of involvement and are reported in 60% to 90% patients with or without involvement of other lymphoid tissue [21]. Cervical lymphadenitis, also known as scrofula, may be a manifestation of a systemic tuberculous disease or a unique clinical entity localized to the neck.

Classically, patients with TBLN showed symptoms such as associated fever, weight loss, and fatigue and somewhat less frequently with night sweat. Authors have reported that clinical symptoms are a prominent feature associated with *M. tuberculous* lymphadenitis [22]. In our study, we also observed that weight loss (73%) was a common clinical symptom followed by fever (72%) in the patients of TBLN. However, the presence of night sweat was observed less frequently in the study, which showed similarity with the findings in the literature [22–24].

In our study, Anorexia was observed in 66% patients in comparison to 46% reported in a study by Verma et al. [25]. This might be linked to the lack of access to health services, economical factors, and the awareness among the patients in the study area. Lee et al. reported that 57% had no clinical symptoms [26], while in the present study, 11% of patients had no clinical symptoms which may be due to the asymptomatic progression of the disease and the patients' early stages of the disease.

Polymerase chain reaction (PCR) testing directly from clinical samples has revolutionised diagnosis in many areas of microbiology. However, PCR can improve the diagnosis of extrapulmonary tuberculosis, especially TBLN, from the aspirate samples. PCR is a fast and useful technique for the demonstration of mycobacterial DNA fragments in patients with clinically suspected TBLN [27]. The FNA PCR positivity rate of 40% to 90% has been reported in cases of TBLN [27]. In our study, 48% cases were positive by PCR, which is more than the positivity reported by various studies [12, 28–30]. This may be due to the fact that, in the present study, there might be sufficient numbers of DNA copies available in the specimens.

The sensitivity of PCR have been reported between 43% and 84%, and its specificity between 75% and 100% [31]. PCR sensitivity was found to be 83% and specificity to be 100% in diagnosing TBLN with FNAC [32]. In our study, the sensitivity and specificity of PCR was found to be 83.3% and 61.5% respectively, which was found to be in concordance with a study reported sensitivity of 87.4% and specificity of 66.7% [25]. In the present study, the sensitivity and specificity of PCR when compared with culture was 93.75% and 100%, respectively.

In our study, the positivity of PCR was 100% in specimens positive by both AFB smear and culture and 21.2% in culture-negative specimens, with an overall positivity of 48%. Our result was comparable with the study reported from India which indicated 100% PCR positivity in AFB smear and culture-positive specimens [33]. The results indicate that PCR was a useful and highly sensitive technique in the diagnosis of TBLN in suspected cases [34].

On the other hand, 14 (21.2%) culture-negative and smear-negative samples were also detected by PCR assays. This indicated that by using combined conventional methods, cytology and PCR, it is possible to enhance the detection rate and isolation of etiological agents of TBLN. A possible explanation for the success of the PCR assay relative to the conventional methods is that the PCR assay is more sensitive than the conventional methods, particularly when low numbers of bacilli are present.

## 5. Conclusions

In our study, we observed that PCR was able to diagnose the additional cases, which were negative by direct smear microscopy, LJ & BACTEC culture, and suggested that TBLN along with other cases, have no clinical symptoms. PCR positivity was observed at 100% in direct smear microscopy and culture-positive specimens which suggested that PCR is a rapid, efficient, sensitive, and specific technique for the diagnosis of TBLN cases [35].

## Figures and Tables

**Table 1:** Comparative analysis of direct smear microscopy, culture by LJ & BACTEC and PCR (*n* = 100).

Serial number	Methods	Positive (%)	Negative (%)	Total number
1	Direct smear microscopy positive	24 (24%)	76 (76%)	100
2	Culture by LJ	34 (34%)	66 (66%)	100
3	Culture by BACTEC	45 (45%)	55 (55%)	100
4	PCR	48 (48%)	52 (52%)	100

## Data Availability

The data used to support the findings of this study are included in the article.
